# Melanophages give rise to hyperreflective foci in AMD, a disease-progression marker

**DOI:** 10.1186/s12974-023-02699-9

**Published:** 2023-02-08

**Authors:** Sebastien Augustin, Marion Lam, Sophie Lavalette, Anna Verschueren, Frédéric Blond, Valérie Forster, Lauriane Przegralek, Zhiguo He, Daniel Lewandowski, Alexis-Pierre Bemelmans, Serge Picaud, José-Alain Sahel, Thibaud Mathis, Michel Paques, Gilles Thuret, Xavier Guillonneau, Cécile Delarasse, Florian Sennlaub

**Affiliations:** 1 Sorbonne Université, INSERM, CNRS, UMR_S 968, Institut de la Vision, 17 rue Moreau, 75012 Paris, France; 2 Ophthalmology Department, Université de Paris, APHP, Hôpital Lariboisière, 75010 Paris, France; 3 Cellules Souches et Radiations, Stabilité Génétique, Université de Paris, Université Paris-Saclay, Inserm, CEA, Fontenay-Aux-Roses, France; 4 Laboratoire des Maladies Neurodégénératives, Université Paris-Saclay, CEA, CNRS, MIRCen, Fontenay-Aux-Roses, France; 5 Centre Hospitalier National d’Ophtalmologie des Quinze-Vingts, INSERM-DHOS CIC 503, Paris, France; 6 Service d’Ophtalmologie, Hôpital de la Croix-Rousse, Hospices Civils de Lyon, UMR CNRS 5510 MATEIS, Université Lyon 1, 103 Grande rue de la Croix Rousse, 69317 Lyon Cedex 04, France; 7 Laboratory of Biology, Engineering and Imaging for Ophthalmology, BiiO, EA2521, Faculty of Medicine, University of Saint Etienne, Saint Etienne, France

**Keywords:** Age-related macular degeneration, Macrophage, CD47, Neuroinflammation

## Abstract

Retinal melanosome/melanolipofuscin-containing cells (MCCs), clinically visible as hyperreflective foci (HRF) and a highly predictive imaging biomarker for the progression of age-related macular degeneration (AMD), are widely believed to be migrating retinal pigment epithelial (RPE) cells. Using human donor tissue, we identify the vast majority of MCCs as melanophages, melanosome/melanolipofuscin-laden mononuclear phagocytes (MPs). Using serial block-face scanning electron microscopy, RPE flatmounts, bone marrow transplantation and in vitro experiments, we show how retinal melanophages form by the transfer of melanosomes from the RPE to subretinal MPs when the “don’t eat me” signal CD47 is blocked. These melanophages give rise to hyperreflective foci in Cd47^−/−^-mice in vivo, and are associated with RPE dysmorphia similar to intermediate AMD. Finally, we show that Cd47 expression in human RPE declines with age and in AMD, which likely participates in melanophage formation and RPE decline. Boosting CD47 expression in AMD might protect RPE cells and delay AMD progression.

**Supplementary Information:**

The online version contains supplementary material available at 10.1186/s12974-023-02699-9.

## Introduction

Age-related macular degeneration (AMD) affects more than 150 million people worldwide (early AMD) and 10 million patients suffer from debilitating late stage AMD [1, 2]. Early/intermediate AMD is characterized by pigmentary changes and lipoproteinaceous debris accumulation between the photoreceptors and the melanosome-rich retinal pigment epithelium (RPE, pseudodrusen) or below the RPE (soft drusen). Later, AMD can be complicated by central choroidal neovascularization (neovascular AMD, late form) and ultimately a disciform scar (neovascular AMD end stage), or by an extending lesion of the photoreceptors, RPE, and choroid that often starts parafoveally (geographic atrophy, GA, late form) [3].

Patients with early/intermediate AMD can progress and develop late AMD (~ 15% in the Beaver Dam study over 10 years; ~ 30% in the Blue mountain study over 6 years), but a large part of patients stay stable for years [4, 5], underlining the potential usefulness of progression biomarkers of AMD. Recently, retinal imaging by spectral-domain optical coherence tomography (SD-OCT) identified hyperreflective foci (HRF), as a highly predictive biomarker for progression from intermediate to late AMD [6, 7]. HRF are defined as discrete, well-circumscribed intraretinal lesions with reflectivity comparable to the retinal pigment epithelium (RPE) band on SD-OCT [6, 7]. Their presence is also associated with the two major genetic AMD risk factors the CFH H402 variant and a 10q26 haplotype [8].

In a direct comparison study of post-mortem SD-OCT and histology of drusenoid pigment epithelium detachment (a known precursor to GA), HRF were identified to be caused by melanosome/melanolipofuscin-containing sub- and intra-retinal cells (MCCs) [9]. As MCCs occur in intermediate AMD before major RPE death occurs and the RPE is the only melanin containing cell type under the healthy retina, it is widely believed that HRF are caused by migrating retinal pigment epithelium [2, 9]. However, a direct comparison of SD-OCT and immunohistochemistry of a laser lesion in a human patient revealed that HRFs co-localized with MPs [10] and immunohistological studies of MP distribution in AMD, revealed the presence of pigment-containing MPs [11, 12], raising the possibility that MPs that ingested melanosomes/melanolipofuscin can be MCCs and by extension the anatomical equivalent of HRF and a bad prognostic factor in AMD.

MPs are a family of cells that include monocyte (Mo), resident macrophages (rMφ) such as microglial cells (MC), and monocyte-derived inflammatory macrophages (iMφ) that arise during inflammation [13]. Their accumulation has been shown to play an important role in the pathogenesis of many chronic, age-related diseases [13, 14], including late AMD [3, 10] where they have been shown to play a critical role in neovascularization and photoreceptor degeneration [3]. Importantly, MP accumulation is also observed around reticular pseudodrusen [15] and large drusen that characterize early/intermediate AMD [11, 16]. At this earlier stage they might fulfill a homeostatic role, controlling debris accumulation, or provoke further degeneration, possibly depending on the patients AMD risk factors that determine the MPs pathogenic potential [3].

We recently showed that the homeostatic elimination of infiltrating MPs is dependent on Thrombospondin 1 (TSP1)-mediated activation of the CD47 receptor and Thbs1^−/−^- and Cd47^−/−^-mice develop age-related subretinal MP accumulation [17]. We demonstrated that both major genetic AMD risk factors, the CFH H402 variant and a 10q26 haplotype, inhibit TSP1-mediated CD47 activation and MP elimination, promoting pathogenic inflammation [17, 18]. Independently of TSP1, CD47, expressed on many cell types, also functions as the ligand for signal regulatory protein α (SIRPα) [19]. SIRPα is expressed on all myeloid cells, including monocytes, macrophages and microglia, and its ligation by CD47 induces a “don’t eat me” signal inhibiting the myeloid cell-mediated removal of the CD47-expressing cell [19].

Together, these observations raise the question whether melanosomes/melanolipofuscin particle-containing MPs can represent the underlying anatomical structures of HRF and how MPs ingestion of RPE melanosomes/melanolipofuscin particle affects RPE homeostasis.

Using human AMD sections, we here show that intraretinal pigment, internally to the RPE cell layer, is never found in cells positive for RPE or macroglial cell markers but locates to melanin/melanolipofuscin-laden macrophages previously described as melanophages in hyperpigmentation disorders or melanotic lesions of the skin [20–22]. Using CD47^−/−^-mice and CD47 blocking antibodies, we demonstrate that the lack of the “don’t eat me signal” on RPE is sufficient to generate subretinal melanophages, associated with RPE dysmorphia, strikingly similar to intermediate AMD lesions. Last but not least, we show that Cd47 expression declines in human RPE with age and in AMD patients, which likely participates in melanophage formation and associated RPE deterioration in AMD.

## Methods

### Immunohistochemistry on donor eyes sections

Four control donor eyes and 12 donor eyes of 11 donors with a known history of AMD, melanosomes/melanolipofuscin-containing cells visible in unstained bright-field microscopy, and histological evidence for intermediate AMD (sizeable drusen), neovascular AMD (subretinal presence of vessels without gliosis), disciform scar (subretinal presence of vessels with gliosis), or GA (lesions with complete outer retinal and RPE atrophy) were used in this study (see Table 1).

Informed consent was obtained for all donors by the Minnesota Eye bank and experiments conformed to the principles set out in the WMA Declaration of Helsinki. The death to ocular cooling time and the death to enucleation time comprised between 45 min-6h45min and 2h45 and 7h15, respectively. The posterior segment was fixed 4 h in 4% paraformaldehyde. 8-μm horizontal sections of paraffin embedded human tissues crossing the optic nerve and perifovea were cut with a microtome (Microm Microtech France). The sections were de-paraffinized by 30 min incubation in QPath® Safesolv (VWR Chemicals) and rehydration was performed in 5-min serial baths of alcohol (100/95/70%) and water. Antigen retrieval was performed in boiling citrate buffer pH6 for 20 min. Sections were blocked with 1% horse serum 30 min and then exposed overnight to recombinant rabbit monoclonal anti-RPE65 (ab231782, 1:200, Abcam), rabbit anti-human peropsin (LS-A1150, 1:200, LSBio), mouse anti-human CD68 (NCL-L-CD68, 1:40, Leica Biosystems), mouse anti-human CD163 (NCL-L-CD163, 1:25, Leica Biosystems), rabbit anti-IBA1 (019-19741, 1:200, Fujifilm Wako), mouse anti-GFAP (G3893, 1:200, Sigma-Aldrich) antibodies. After washing, sections were incubated with appropriate secondary goat anti-rabbit or goat anti-mouse antibody conjugated to an alkaline phosphatase (1:500, ThermoFisher Scientific) for 60 min, followed by revelation with Fast-Red (Sigma-Aldrich) following the manufacturer instructions. Sections were counterstained with Hoechst 33342 (1:1000, ThermoFisher Scientific). Autofluorescence was observed in the green channel (excitation filter bandpass 470/40 and suppression filter bandpass 525/50). The slides were then washed, mounted, and viewed and photographed with a Leica DM550B fluorescence microscope (Leica Biosystems). The total surface, and immuno-stained surface covered by intraretinal pigment were measured for each retinal pigmented focus for each antibody and the percentage of immune-stained surface of the total pigmented surface was calculated for each eye. Control experiments omitting the first antibody gave no staining (data not shown). Consecutive serial slides were used to carry out stainings with multiple antibodies. Control human tonsil sections processed using the same experimental procedure, were used to validate the antibodies.

### Animals

Wild-type (WT) C57BL/6J control, *Cd47*^−/−^ and *Thbs1*^−/−^ -mice were obtained from Charles River. All mice used in this study were male and were *rd8* mutation free, as this mutation can lead to an AMD-like phenotype. Male mice were used to eliminate the influence of the reproductive cycle. The mice were kept to the indicated ages under specific pathogen-free condition in a 12 h/12 h light/dark (100 lx) cycle with no additional cover in the cage and with water and normal chow diet available ad libitum. All experimental protocols and procedures were approved by the French Ministry of higher Education, Research and Innovation (authorization number #00075.01, #2218 2015090416008740 v4). All procedures were performed under anesthesia and all efforts were made to minimize suffering.

### Optical coherence tomography (OCT) imaging in mice

Pupils were dilated with tropicamide (Mydriaticum, Théa, France) and phenylephrine (Neosynephrine, Europhta, France). Animals were then anesthetized by intraperitoneal injection of ketamine (50 mg/kg) and xylazine (10 mg/kg). SD-OCT images were taken with the SD-OCT imaging device (Bioptigen 840 nm HHP; Bioptigen, North Carolina, USA). Eyes were kept moisturized with 9‰ NaCl during the whole procedure. Image acquisitions were performed on Bioptigen acquisition software and processed with open source Fiji software (http://fiji.sc/Fiji).

### Immunohistochemistry on mice eye sections

Mice were killed by CO2 asphyxiation and eyes were enucleated. Eyes were fixed for 1 h in 4% PFA, then rinsed and sectioned at the limbus; the cornea and lens were discarded. Eyecups were incubated in 30% sucrose overnight at 4 °C, then embedded in OCT and sectioned (10 µm). Cryosections were blocked with PBS containing 1% horse serum, 0.1% Triton 1 h at room temperature and exposed overnight to rabbit anti-IAB1 antibody (019-19741, 1:200, Fujifilm Wako) at 4 °C. After washing, sections were incubated 2 h with an Alexa Fluor 488-conjugated donkey anti-rabbit IgG (1:500, ThermoFisher Scientific) and counterstained with Hoechst 33342 (1:1000, ThermoFisher Scientific). The slides were then washed, mounted, and viewed and photographed with a Leica DM550B fluorescence microscope (Leica Biosystems).

### MP and RPE quantification on mouse RPE/choroidal flatmounts

Mice were killed by CO2 asphyxiation and eyes were enucleated. The globes were fixed in 4% PFA for 45 min, and then sectioned at the limbus; the cornea and lens were discarded. RPE/choroid tissues were separated from retina and incubated overnight with rabbit anti-IBA1 antibody (019-19741, 1:400, Fujifilm Wako) and Alexa Fluor 594 phalloidin (1:100, ThermoFisher Scientific) in PBS containing 0.1% Triton. Tissues were rinsed and incubated 2 h with an Alexa Fluor 488-conjugated donkey anti-rabbit IgG (1:500, ThermoFisher Scientific) and counterstained with Hoechst 33342 (1:1000, ThermoFisher Scientific). RPE/choroids were flatmounted, viewed and photographed with a Leica DM550B fluorescence microscope (Leica Biosystems). MPs were counted on whole RPE/choroidal flatmounts. RPE nucleation and morphology were evaluated on randomized photos taken between optic nerve and the mid-periphery retina. Melanophages on RPE/choroidal flatmounts were defined and quantified as IBA1^+^ MPs (green fluorescence) that visibly block the red Alexa Fluor 594-phalloidin fluorescence of the underlying RPE when viewed in the red channel.

### Retinal flatmount preparation with adherent RPE

Mice were killed by CO2 asphyxiation and eyes were enucleated. The globes were transferred into PBS solution without calcium. After cleaning from excess of tissues around the sclera, eyes were incubated 40 min in a solution containing L-cysteine (0,035 mg/ml in PBS) and 10 unit of Papain (Worthington) at 37 degrees Celsius, then transferred in DMEM containing 10% fetal bovine serum for dissection; the cornea was first removed by carefully cutting along the ora serrata, the choroid with sclera was delicately detached by peeling until the optic nerve. Finally, the lens and iris were removed by cutting around. Retinal/RPE tissues were then fixed in PFA 4% for 45 min and then rinsed with PBS and flatmounted and scanned with the Hamamatsu Nanozoomer Digital Pathology (NDP) 2.0 HT (Hamamatsu Photonics, France).

### Serial block-face scanning electronic microscopy

Mice were killed by CO2 asphyxiation and eyes were enucleated. The globes were fixed in PBS containing 2% paraformaldehyde, 1% glutaraldehyde during 1 h at room temperature. Samples were then prepared for Serial Block Face using the NCMIR protocol (https://ncmir.ucsd.edu/sbem-protocol). They were post-fixed for 1 h in a reduced osmium solution containing 1% osmium tetroxide, 1.5% potassium ferrocyanide in PBS, followed by incubation with a 1% thiocarbohydrazide (TCH) solution in water for 20 min at room temperature. Subsequently, samples were fixed with 2% OsO_4_ in water for 30 min at room temperature, followed by 1% aqueous uranyl acetate at 4 °C overnight. The samples were then subjected to en bloc Walton’s lead aspartate staining and placed in a 60 °C oven for 30 min. Then samples were dehydrated in graded concentrations of ethanol for 10 min each. The samples were infiltrated with 50% Agar low viscosity resin (Agar Scientific Ltd) overnight. The resin was then changed and the samples further incubated during 3 h prior to inclusion in returned capsules and polymerized for 18 h at 60 °C. The polymerized blocks were mounted onto special aluminum pins for SBF imaging (FEI Microtome 8 mm SEM Stub, Agar Scientific), with two-part conduction silver epoxy kit (EMS, 12642-14). Samples mounted on aluminum pins were trimmed and inserted into a TeneoVS SEM (ThermoFisher Scientific). Acquisitions were performed with a beam energy of 2 kV, a current of 100pA, in HiVac mode with the filtering system, a dwell time of 1 µs per pixel and sections of 50 nm. The pixel size was 10 nm. Images were processed for 3D reconstitution and segmentation using Imaris software (Oxford Instruments).

### Human RPE and monocytes co-culture

Human RPE (ARPE-19) cells (ATCC) were seeded in 48-well plate and cultured 10 days in DMEM/F12 medium (ThermoFisher Scientific) supplemented with 10% heat inactivated fetal bovine serum (ThermoFisher Scientific) and 1% (v/v) penicillin (100 U/ml)/streptomycin (100 μg /ml) (ThermoFisher Scientific) at 37 °C with 5% CO2. Cells were serum-starved 24 h before experiment and labeled with FarRed CellTrace (ThermoFisher Scientific) following manufacturer instructions. Human blood monocytes from healthy donor were purified after written and informed consent in the Centre National d’Ophtalmologie des Quinze-Vingts (Paris, France). Briefly, peripheral blood mononuclear cells were isolated from blood by Ficoll gradient centrifugation and monocytes were isolated using EasySep Human Monocyte Enrichment Kit (StemCell Technologies). 300 000 freshly purified human monocytes were added to confluent ARPE-19 cells (1:1 ratio) in DMEM containing 1% penicillin/streptomycin with 10 µg/ml of mouse anti-human CD47 blocking antibody (B6H12) (14-0479-82, eBioscience) or mouse IgG Isotype Control (eBioscience). After 2 h incubation cells were treated with Accutase (ThermoFisher Scientific) and then washed with PBS. Cells were labeled with FITC-conjugated CD14 antibody (clone REA599, Miltenyi Biotec). Cell-bound antibodies and FarRed CellTRace were detected with a FACSCelesta analyzer (BD Biosciences) and the data were analyzed with FlowJo Software (FlowJo, LLC).

### Bone marrow transplantation

Twenty-four hours before transplantation 6-month-old WT and *Cd47*^−/−^ recipient mice were lethally irradiated with 10 Gy (1 Gy/min) of total body irradiation from a ^137^Cs source. Bone marrow cells were collected from the tibias and femurs of age-matched wild-type mice, rinsed and resuspended in PBS. Recipient mice were intravenously injected with 3 × 10^6^ bone marrow cells from donors via the tail vein. 6 months after bone marrow transplantation, mice were killed and the eyes were enucleated for MP/melanophage quantification on RPE/choroid flatmounts.

### CD47 expression in human tissues

The human ocular tissues used for CD47 expression analysis in this study were obtained from body donation for science, handled in accordance with the Declaration of Helsinki. Each donor had volunteered their body and had provided written consent to the Laboratory of Anatomy of our Faculty of Medicine, Saint-Etienne, France). After removal of the anterior segment through a circular incision at the equator and delicate removal of the neuroretina, 350 µl of RA1 buffer (Macherey Nagel) were added to the posterior segment, covering the central exposed RPE cells. After 5 min, the buffer containing the lysed RPE cells were pipetted up and down 5 times. The lysates were then processed following supplier instructions. Single-strand cDNA was synthesized with 1 µg of RNA pretreated with DNase amplification grade, using oligo-dT as primer and Superscript II reverse transcriptase (Thermo Fisher Scientific). For real-time PCR, 1/100 of cDNA was incubated with the polymerase and the appropriate amounts of nucleotides (TaqMan Gene Expression Master Mix, Applied Biosystems; Power SYBR Green PCR Master Mix, Applied Biosystems). qPCR were realized with the QuantStudio real-time PCR system (Applied Biosystems) using the following parameters: 45 cycles of 15 s at 95 °C, 45 s at 60 °C. Results were normalized with expression of RPS26 as an housekeeping gene.

RPS26_S: TCGATGCCTATGTGCTTCCC; RPS26_S: TCGATGCCTATGTGCTTCCC; CD47 TaqMan® Assays Hs00179953_m1.

RPE normalized CD47 expression in the transcriptome dataset of RPE/choroid samples of Newman et al.

Data from Newman et al. [23] from the GEO database (https://www.ncbi.nlm.nih.gov/geo/query/acc.cgi?acc=GSE29801) were downloaded and the expression data of the RPE/choroid samples were downloaded. We further filtered the samples to keep only patients older than 60 and analyzed the data from the “normal” samples (controls, *n* = 36) and from intermediate AMD patients (*n* = 18) classified in Newman et al. as “MD2” (*n* = 4; soft distinct drusen > 63 µm/pigmentary changes) and “dry AMD” (*n* = 14; soft indistinct drusen > 125 µm, reticular drusen, soft distinct drusen in association with pigmentary changes, soft distinct drusen in association with pigmentary changes). To normalize the expression data of RPE/choroid samples for the content of RPE, we filtered 40 RPE-specific transcripts from the single cell transcriptomic dataset GSE135922 (https://www.ncbi.nlm.nih.gov/geo/query/acc.cgi?acc=GSE135922) [24] a) among the 200 transcripts most expressed in RPE compared to choroidal cells and b) that were specific for RPE cells ( SLC39A12,SLC22A8,SLC2A12,SLC6A13,SLC6A20,PLD5,ERMN,BMP7,PNPLA3,MYRF,STRA6,SLC16A8,SLC4A5,RP11.509E16.1,STRIP2,CHRNA3,CSPG5,GPM6A,LINC00982,VAT1L,CLIC6,TMEM56,RGR,LRAT,RPE65,RLBP1,PLA2G5,SFRP1,TRPM3,C1orf61,MFAP3L,RDH5,IFITM10,TPRN,ALDH1A3,FAM221A,APLP1,SOX9,OTX2,KCNJ13,MT1G). CD47 expression for each of the GSE29801 data points were normalized for the mean of the 40 RPE-specific genes in each sample.

## Results

### Intraretinal pigment in AMD is primarily located in melanophages

**Fig. 1:**
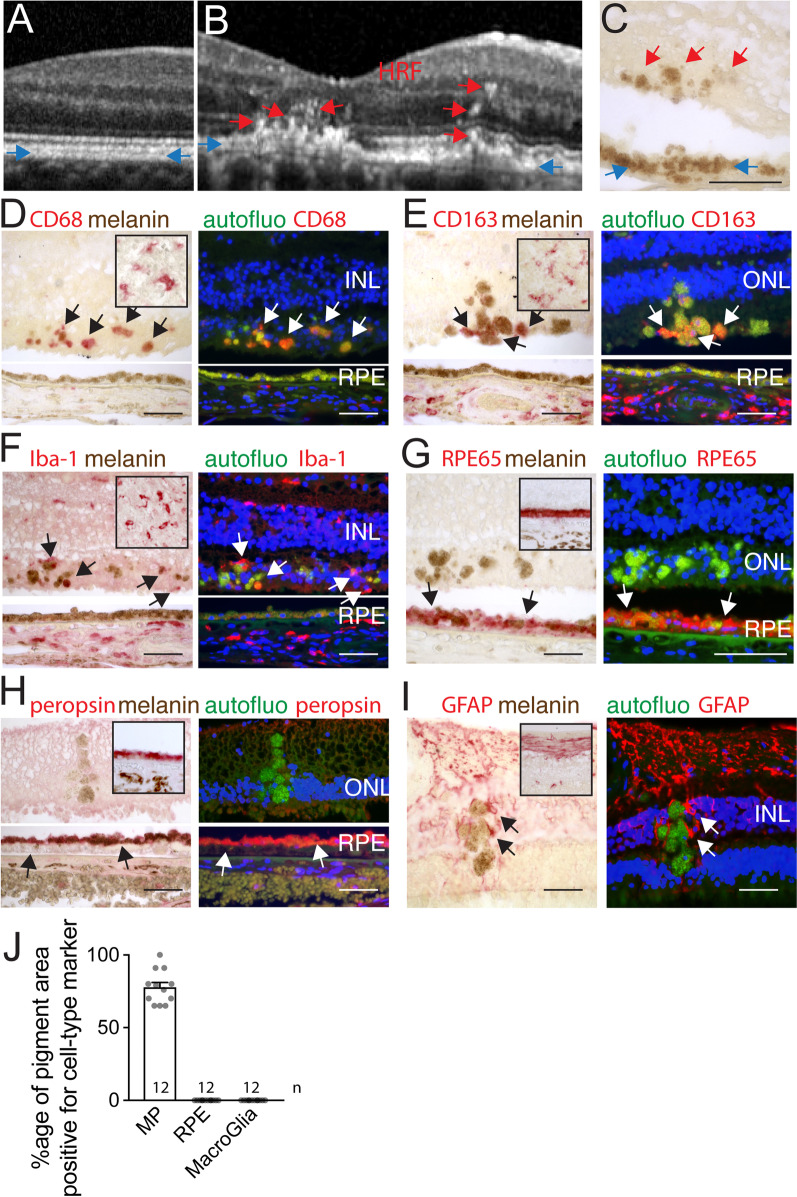
Intraretinal pigment in AMD is primarily located in melano-macrophages. RPE hyperreflective band (blue arrows) and hyperreflective foci (HRF, red arrows) visualized by SD-OCT of the retina of a healthy subject (**A**) and a patient with intermediate AMD (**B**). The aspect of the RPE (blue arrows) and retinal pigmented foci (red arrows) in an unstained paraffin section adjacent to the atrophic lesion of an AMD donor (**C**). CD68 (**D**), CD163 (**E**), IBA1 (**F**), RPE65 (**G**), peropsin (**H**), GFAP (**I**) staining in bright-field and fluorescence microscopy, of tonsils (insets **C**-**F**), healthy control retina (insets **G**-**I**) and retinal pigmented foci and adjacent RPE (**C–I**). The signal was revealed using Fast red chromogenic substrate visible in red in bright-field and in the red channel in fluorescence microscopy (arrows), autofluorescence was captured in the green channel and Hoechst nuclear stain in the blue channel. Immunohistochemistry experiments omitting the primary antibody served as negative controls (not shown). Calculation of the percentage of surface covered by immuno-stained retinal pigmented foci of total retinal pigmented foci for each immunostaining in each of the 12 donor eyes (**J**). *HRF* hyperreflective foci, *RPE* retinal pigment epithelium, *INL* inner nuclear lacer, *ONL* outer nuclear layer, scale bar = 50 µm; All values are reported as mean ± SEM

The high content of melanosomes of the healthy retinal pigment epithelium (RPE) is an important contributor for its appearance as a single hyperreflective band in spectral-domain optical coherence tomography (SD-OCT), a clinically used method to visualize the retina and choroid, shown here is an example of a healthy individual (blue arrows Fig. 1A). In patients with intermediate AMD, hyperreflective foci (HRF), defined as discrete, well-circumscribed lesions with a reflectivity similar to the RPE can regularly be observed sub- and intra-retinally, shown here in a patient with intermediate AMD (red arrows Fig. 1B). HRF have been shown to be a highly predictive biomarker for progression from intermediate to late AMD [6, 7, 25] and they are also more common in AMD carriers of the main genetic risk factors [8]. In unstained histological sections of AMD patients, foci of pigmentation that resembles the RPE pigment is regularly observed internally to the RPE monolayer (Fig. 1C). These melanin containing cells (MCCs) are generally believed to be migrating RPE cells and start occurring in intAMD before major RPE cell death occurs.

To identify the cell types that constitute the MCC-pool, we performed immunostaining on paraffin sections of 12 eyes of 11 donors with a known history of AMD that all contained MCCs, visible by unstained bright-field microscopy (example Fig. 1C). The histological sections of one donor additionally had sizeable drusen without atrophy or CNV, seven donors had visible atrophic lesions on post-mortem fundus examination and in histology, sections from two donors featured subretinal CNV without gliosis, and three donors were characterized by a disciform scar on fundus examination and in histology (Table 1). MPs were detected by CD68 (Fig. 1D), CD163 (Fig. 1E), and ionized calcium-binding adapter molecule 1 (IBA1, Fig. 1F) staining, the RPE by Retinal pigment epithelium-specific 65 kDa protein (RPE65, Fig. 1G) and peropsin (Fig. 1H) staining; and macroglia by glial fibrillary acidic protein (GFAP, Fig. 1I) immunohistochemistry. Healthy eye donors (inset Fig. 1G–I) and human tonsils (inset Fig. 1D–F) served as positive controls. We used a chromogenic substrate revealing method (alkaline phosphatase/Fast Red) that is visible in bright field and in red fluorescence and observed autofluorescence in the green channel (excitation filter bandpass 470/40 and suppression filter bandpass 525/50) and a Hoechst nuclear marker in the blue channel. Bright field photographs of immune staining of CD68, CD163 and IBA1, revealed a red staining in the typical form and location of MPs in stained tonsil sections (inset Fig. 1D–F) and choroid (where CD68 only stains a subset of choroidal macrophages as previously reported [12]) but no red staining was detected in RPE, identified as the typical monolayer of pigmented cells, demonstrating the MP specificity of the staining. Observation of retinal pigmented foci revealed that a substantial portion of the pigmented intraretinal structures appears red (positive for the FastRed pigment used to reveal the immunohistochemistries) for each of the MP markers (arrows, Fig. 1D–F) compared to their brown color of unstained sections (Fig. 1C), to negative controls omitting the primary antibody (not shown), and to retinal pigmented foci in sections stained for RPE and glial markers (Fig. 1G–I). Accordingly, micrographs/images of fluorescence microscopy, taken with the same exposure times for pigmented foci and the RPE, reveals a strong red component in the foci (red fluorescence and red/green double positive fluorescence appearing as yellow), compared to the green only autofluorescence emanating from the RPE of the same sections (Fig. 1D–F), and to pigmented foci stained for RPE and glial markers (Fig. 1G–I). Immunostaining for RPE65 and peropsin, two RPE-specific proteins, stained the RPE mono-layer of healthy control donors red in bright-field observation (insets Fig. 1G and H) and strongly marked the RPE in AMD sections visible in bright field and in red fluorescence, but failed to stain any pigmented foci of the retina (Fig. 1G and H). Similarly, GFAP staining revealed a staining in an astrocyte distribution in healthy controls (inset Fig. 1I) and a staining pattern typically observed in activated Müller cells in AMD sections, but the staining never overlapped with pigmented foci, despite coming very close (Fig. 1I). We next measured the surface of the immunohistochemistry sections that contained melanosomes/melanolipofuscin particles and the surface that was additionally positive for the immunostaining and calculated the percentage of the surface of the retinal sections that was double positive over the total surface containing melanosomes/melanolipofuscin granules for each immunostaining in each of the 12 donor eyes (Fig. 1J). Strikingly, retinal pigmented foci were never found to be positive for either RPE or macroglial cell markers in any of our donor eyes. However, we found that between 70 and 100% of pigmented foci surface was positive for at least one of the MP markers in each of the patients. As we were technically unable to simultaneously stain for all three MP markers, we do not know whether MP-marker negative pigmented foci with one staining would have stained positive for one of the other used MP markers (or markers not used in this study) and it is additionally possible that not all retinal pigment is located intracellularly. Interestingly, in sections of two patients with disciform scars, a substantial number of pigmented cells found within the scar (but not in the retinal parenchyma) stained in part positive for RPE65 and peropsin (data not shown), suggesting that islands of RPE cells have become surrounded by the scar tissue on certain sections.

**Table 1:** Human cases analyzed in immunohistochemistry

Eye no.	Age	Sex	Type of AMD	Ophthalmologic history	Medical history
1	95	F	Intermediate AMD	Cataract	Hypertension, stroke, tobacco
2	85	M	GA	Cataract	Cardiopathy, dyslipidemia, tobacco
3	81	F	GA		Hypertension, dyslipidemia, renal failure
4	90	F	GA	Cataract	Hypertension, dyslipidemia, cardiopathy, renal failure, liver cancer
5	90	F	GA	Cataract	Hypertension, dyslipidemia, cardiopathy, renal failure, liver cancer
6	86	F	GA		Cardiopathy, renal failure
7	80	F	GA	Cataract	Dyslipidemia, tobacco
8	80	F	GA		Cardiopathy, chronic obstructive lung disease, tobacco, arthritis
9	86	F	Neovascular AMD		Tobacco, lung cancer
10	86	F	Neovascular AMD		Tobacco, lung cancer
11	89	F	DS	Cataract	
12	62	M	DS		Hypertension, Tobacco

*GA* geographic atrophy, *DS* disciform scar

Taken together, our data show that retinal MCCs found in AMD never stain positive for macroglial or RPE cell-specific marker, but the majority can be stained with specific MP markers. Our results confirm a recent report of CD68, CD163 and RPE65 immunohistochemistry [26]. As MPs have been shown to infiltrate the diseased retina in intermediate and late AMD [3, 12, 15, 16, 27], these results strongly suggest that the majority of HRF in AMD are caused by pigment-laden MPs called melanophages that have been well described in skin diseases [20–22].

### Subretinal pigment-laden MPs accumulate in CD47^−/−^mice with age but not in Thbs1^−/−^-mice

**Fig. 2:**
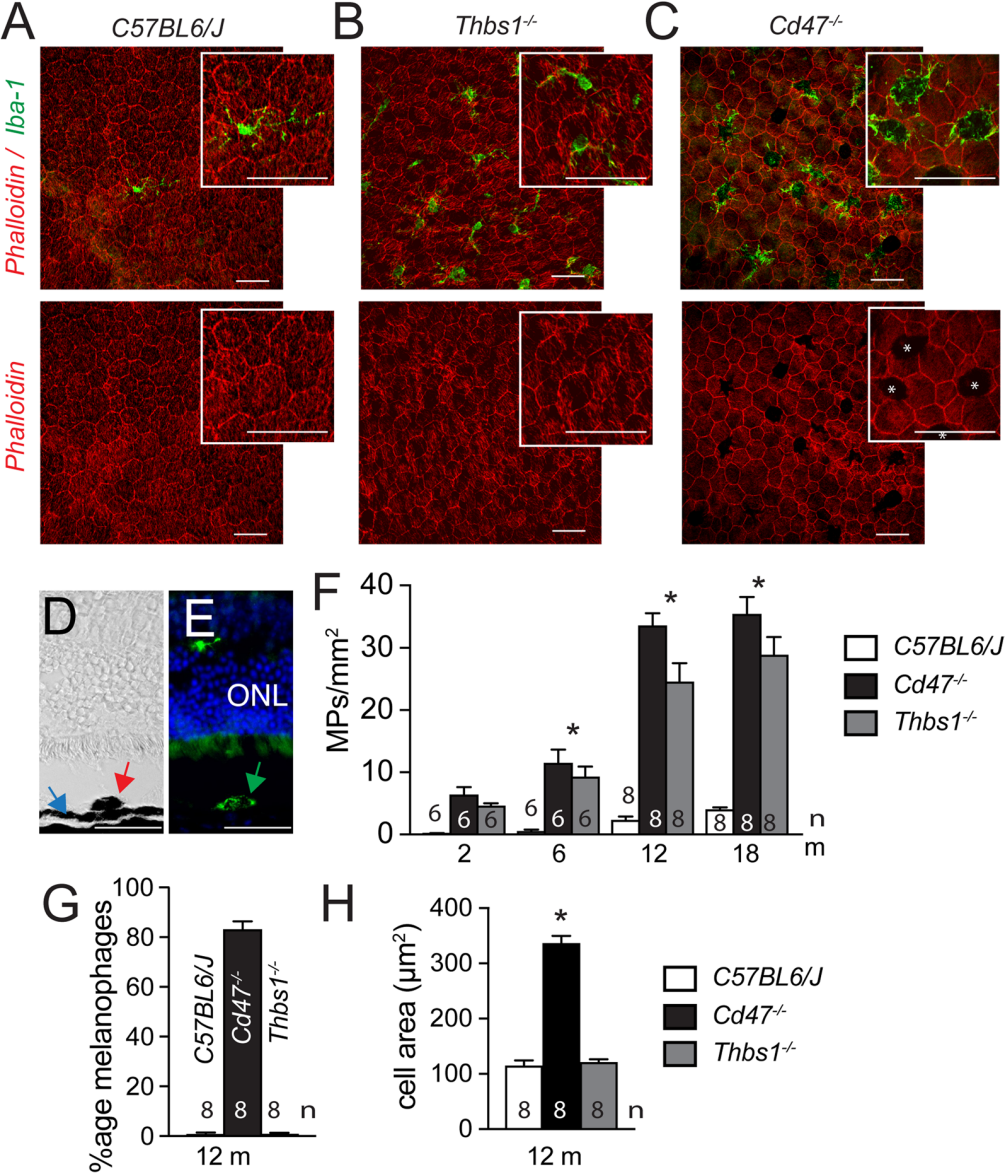
Subretinal pigment-laden MPs accumulate in CD47^−/−^mice with age but not in Thbs1^−/−^-mice. Representative micrographs of phalloidin (red fluorescence staining), IBA1 (green fluorescence staining) double-labeled RPE flatmounts of 12-month-old WT (**A**), Thbs1^−/−^- (**B**) and Cd47^−/−^- (**C**) mice. Asterixis in **C** represent pigment foci that block the phalloidin fluorescence. Representative bright field- (**D**) and fluorescence-microscopy (**E**) views of an anti-IBA1, Hoechst nuclear stain labeled cryo-section of a retinal pigmented focus of a 12-month-old Cd47^−/−^-mouse. Quantification of IBA1 stained subretinal MPs of the indicated mouse strains at the indicated ages (**F**); quantification at 12 months of the percentage of pigment-laden melanophages (that block the Alexa Fluor 594-phalloidin staining of the underlying RPE when viewed in the red channel) of total subretinal MPs (**G**) and the size of the cell body of the subretinal MP expressed as the surface they cover (**H**) on flatmounts (*n* = replicates represent quantifications of eyes from different mice of at least three different experiments and cages; one-way Anova/Kruskal–Wallis test **F** *p = 0,0028 6 m Cd47^−/−^- versus WT-mice; *p* = 0,0324 6 m Thbs1^−/−^- versus WT-mice; *p < 0,0001 12 months Cd47^−/−^- versus WT-mice and 12 months Thbs1^−/−^- versus WT-mice; *p = 0,0019 18 months Cd47^−/−^- versus WT-mice; *p* = 0,0253 18 months Thbs1^−/−^- versus WT-mice; **G** *p < 0.0001 Cd47^−/−^-mice versus WT- and Thbs1^−/−^- mice; **H** *p < 0.0001 Cd47^−/−^-mice versus WT- and Thbs1^−/−^- mice). *Thbs1* Thrombospondin 1 gene, *IBA1* ionized calcium-binding adapter molecule 1, *ONL* outer nuclear layer. Scale bar = 50 µm; All values are reported as mean ± SEM

While melanophage formation in the dermis through ingestion of melanosomes from neighboring melanocytes has been previously described [28], their generation in the retina with an intact RPE cell layer and the mechanisms involved have not been clearly demonstrated. We previously showed that the homeostatic elimination of infiltrating MPs is dependent on Thrombospondin 1 (TSP1)-mediated activation of the CD47 receptor on MPs [17]. Thbs1^−/−^- and Cd47^−/−^-mice therefore develop comparable age-related subretinal MP accumulation contrary to WT mice, confirmed here on phalloidin (red fluorescence staining) and IBA1 (green fluorescence staining) stained RPE/choroidal flatmounts of 12-month-old WT mice (Fig. 2A), Thbs1^−/−^- (Fig. 2B) and Cd47^−/−^-mice (Fig. 2C). However, on closer observation, there is a remarkable difference between the two knockout mouse strains: in Cd47^−/−^-mice the majority of the subretinal IBA1^+^MPs are bloated with a dense pigment which blocks the visualization of the underlying RPE phalloidin stain (red fluorescence; asterixis Fig. 2C), and the IBA1-stain of the MP, which remains visible only at the border and in the dendrites of the MPs. This was not observed in Thbs1^−/−^- mice. IBA1 (green fluorescence) stained cryo-sections of 12-month-old Cd47^−/−^-mice confirmed the presence of pigmented foci (red arrow, Fig. 2D) in the outer retina, internally to the pigmented RPE band (blue arrow, Fig. 2D). These pigmented foci were invariably IBA1-positive (green arrow, Fig. 2E). Quantification of subretinal MPs at 2, 6, 12, and 18 months on IBA1-stained RPE flatmounts, corroborate and extend our previous observation that Thbs1^−/−^- and Cd47^−/−^-mice accumulate subretinal MPs at 12 months, showing the infiltration reaches a plateau from 12 months of age onwards (Fig. 2F). Quantification of subretinal pigment-laden MPs on flatmounts revealed that 80% of all IBA1^+^ subretinal MPs in Cd47^−/−^-mice are filled with pigment to a point that they visibly block the red fluorescence RPE phalloidin staining, when the flatmount is viewed in the red channel. This phenomenon was not observed in WT and Thbs1^−/−^-mice in which the phalloidin RPE staining was continuous and not obscured by over-laying MPs (Fig. 2G). The average size of the bloated subretinal Cd47^−/−^MPs, measured as the area they cover on flatmounts, was tripled compared to control and Thbs1^−/−^-mice (Fig. 2H).

In summary, our study reveals that pigment-containing MPs can form in the retina as seen here in aged Cd47^−/−^-mice similar to dermal melanophages previously described [28], independently of the TSP1-mediated CD47 pathway.

### Massive intracellular accumulation of RPE-derived melanosomes/melanolipofuscin particles in subretinal MPs of CD47^−/−^-mice causes subretinal melanophage formation and their clinical appearance as hyperreflective foci

Melanin, the main pigment found in mammals, is located in melanosomes that are formed in melanocytes and in RPE cells. With age and lipofuscin accumulation the melanosomes can fuse to form melanolipofuscin particles. In the dermis, macrophages ingest melanosomes from neighboring melanocytes to become melanophages, similar to keratinocytes in the epidermis [28]. Subretinal MPs in CD47^−/−^-mice, however, do not have direct contact with melanocytes but with RPE cells, which do not physiologically traffic melanosomes to other cells.

**Fig. 3:**
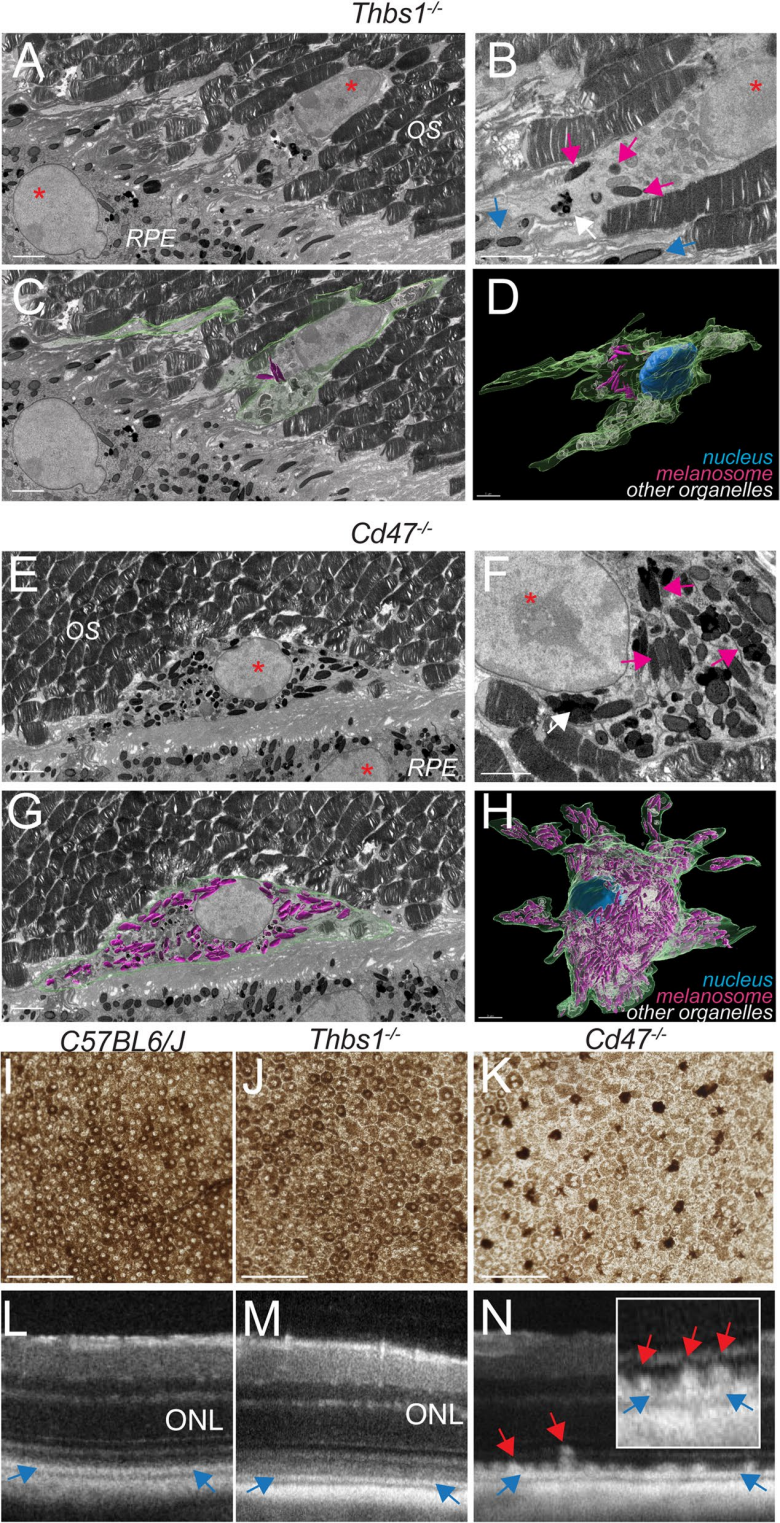
Massive intracellular accumulation of RPE-derived melanosomes in subretinal MPs of CD47^−/−^-mice causes subretinal melanophage formation and their clinical appearance as hyperreflective foci. Representative sections (**A**–**C** and **E**–**G**) and 3D reconstructions (**D** and **F**) of serial block-face scanning electron microscopy (SBF-SEM) of 12-month-old Thbs1^−/−^- (**A**–**D**), and Cd47^−/−^-mice (**E**–**H**). Nuclei are indicated by asterixis, round orthogonally cut and spindle shaped longitudinally cut electron-dense melanosomes are indicated by magenta (in MPs) and blue (in RPE) arrows; melanolipofuscin by white arrows. The border of the subretinal MP (green color), and the surface of each melanosome (magenta) were marked on every SBF-SEM section containing the MPs (**C** and **G**) for the three-dimensional reconstruction of melanosome and melanolipofuscin particle distribution in the subretinal MPs (**D** and **H** and Additional file 1: Movie S1 and Additional file 2: Movie S2; other organelles were marked in white). Representative transmission bright light micrographs of RPE/retinal flatmounts, in which the RPE was kept adherent to the retina of 12-month-old mice of the indicated strains (**I**–**K**). Representative spectral-domain optic coherence tomography images of 12-month-old mice of the indicated strains (**L**–**N**). Blue arrows indicate the RPE hyperreflective line and red arrows indicate retinal hyperreflective lesions. *Thbs1* thrombospondin 1 gene, *OS* outer segments of photoreceptors, *RPE* retinal pigment epithelium. Scale bar = 2 µm

To better define the nature of the pigment that accumulates in subretinal MPs in CD47^−/−^-mice, we performed serial block-face scanning electron microscopy (SBF-SEM) on 12-month-old Thbs1^−/−^- (Fig. 3A–D), and Cd47^−/−^-mice (Fig. 3E–H). Two blocks of each, 12-month-old Thbs1^−/−^-, and Cd47^−/−^-mice, were serially cut until a subretinal MP was captured from beginning to end. Representative images (Fig. 3) show the nuclei of subretinal MPs in the midst of photoreceptor outer segments and adjacent to the RPE nuclei of Thbs1^−/−^-, and Cd47^−/−^-mice (red asterixis Fig. 3A and E). A detailed view of the subretinal Thbs1^−/−^-MP (Fig. 3B) show a melanolipofuscin particle (white arrow) and one orthogonally cut, and two spindle-shaped longitudinally cut, electron-dense melanosomes (magenta arrows), undistinguishable in electron density and shape to RPE melanosomes (blue arrows). Using Imaris imaging software, we next determined the border of the subretinal MP (Fig. 3C; green color), and the surface of all melanosomes and melanolipofuscin granules (magenta) and other organelles such as mitochondria (white) on every SBF-SEM section containing the MP (Additional file 1: Movie S1). The reconstruction of cell reveals that the retinal Thbs1^−/−^-MP contains only very few intracellular melanosomes/melanolipofuscin granules (< 20; Fig. 3D). In contrast, the body of captured subretinal CD47^−/−^-MPs contained densely packed melanosomes (Fig. 3F, magenta arrows) and the three-dimensional reconstruction reveal the extent of melanosome/melanolipofuscin granule accumulation (several hundreds) in the bloated cell body of the CD47^−/−^-MP (Fig. 3 G and H and Additional file 2: Movie S2).

Transmission bright light micrographs of RPE/retinal flatmounts, in which the RPE and retina where kept together using a protocol we usually use for RPE/Retina culture, revealed the densely pigmented RPE in 12-month-old WT- and Thbs1^−/−^- mice (Fig. 3I, J). In contrast, on micrographs of age-matched CD47^−/−^-flatmounts, taken under the same conditions (exposure time, aperture), the RPE’s pigmentation is much diminished and they appear pale in comparison to the densely pigmented subretinal melanophages (Fig. 3K).

From a clinical stand point, while the hyperreflective RPE line in 12-month-old WT-, Thbs1^−/−^-, and Cd47^−/−^-mice was clearly visible in OCT examination (blue arrows Fig. 2L–N), HRFs above the RPE line were only observed in age-matched Cd47^−/−^-mice (red arrows, Fig. 2N), in the exact location and size that retinal melanosomes had been observed (Figs. 2C–E, 3E–H, K), and with striking similarities to HRF observed in AMD (Fig. 1B).

Taken together, these experiments strongly suggest that melanosomes and melanolipofuscin granules from the RPE massively accumulate in subretinal CD47^−/−^-MPs inducing the melanophage phenotype, similar to AMD patients. The fact that subretinal melanophages are visible in Cd47^−/−^-mice as HRF in OCT examination further supports the hypothesis that melanophages are the underlying anatomical features of HRFs in AMD.

### Melanophage accumulation in CD47^−/−^mice is associated with RPE dysmorphia

**Fig. 4:**
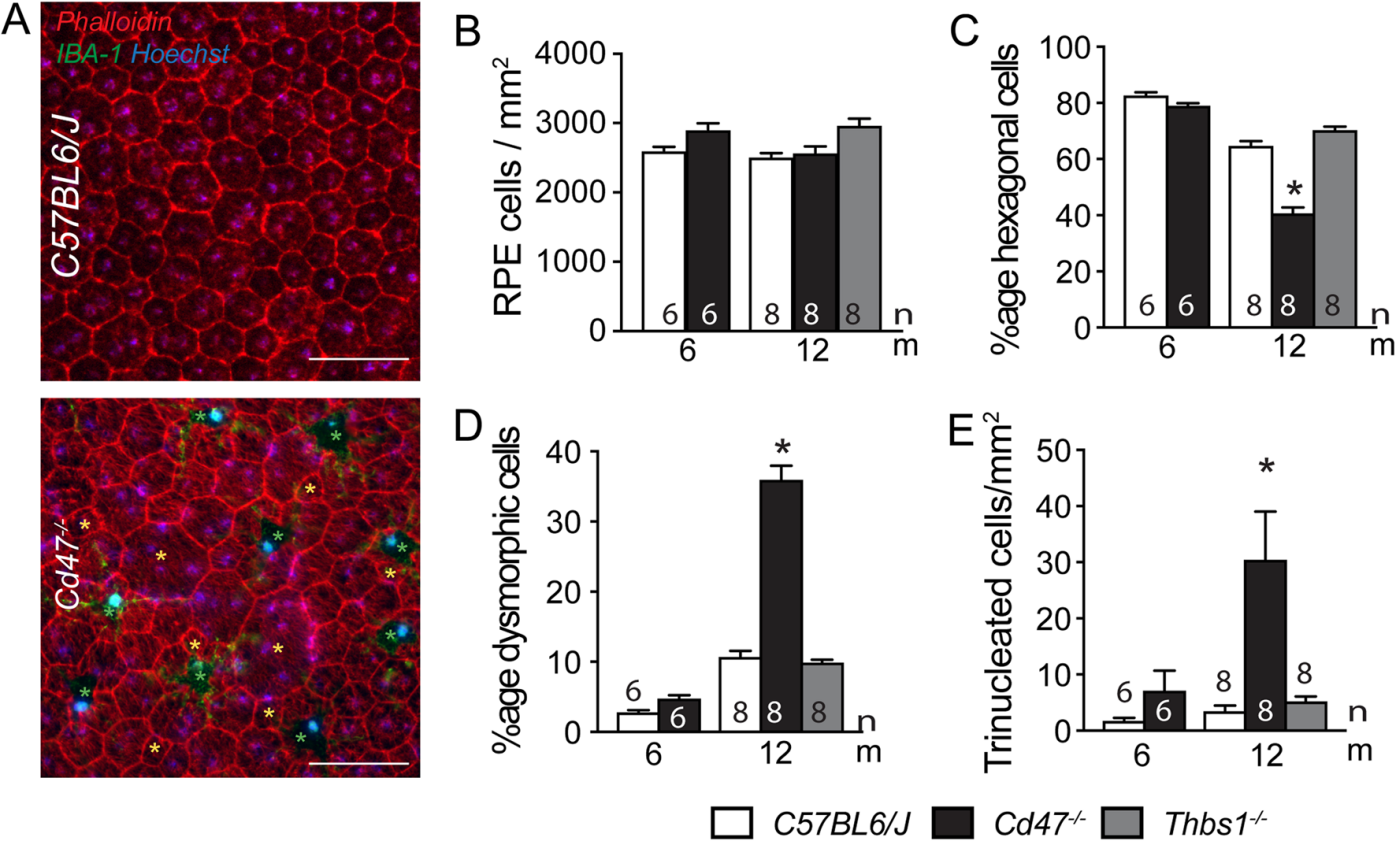
Melanophage accumulation in CD47^−/−^mice is associated with RPE dysmorphia. Representative micrographs of phalloidin (red fluorescence staining), IBA1 (green fluorescence staining) double-labeled RPE/choroidal flatmounts of 12-month-old WT- and Cd47^−/−^-mice (**A**). Green asterixis in indicate melanophages, yellow asterixis indicate dysmorphic RPE cells with less than five or more than six neighbors/sides. Quantification on RPE/choroidal flatmounts of the indicated mouse strains at the indicated ages of RPE cell density (**B**), percentage of hexagonal (**C**), and dysmorphic RPE cells (**D**), and density of trinucleated RPE cells (*n* = replicates represent quantifications of eyes from different mice of at least three different experiments and cages; one-way Anova/Kruskal–Wallis test **C** *p = 0,036 and 0,0004, **D** *p = 0,0041 and 0,0033, and **E** *p < 0.0001 and = 0,9214 12-month-old Cd47^−/−^- versus WT- and Thbs1^−/−^-mice, respectively). *Thbs1* thrombospondin 1 gene, *IBA1* ionized calcium-binding adapter molecule 1; Scale bar = 50 µm

To assess whether melanophages form in CD47^−/−^-mice because subretinal MPs phagocyte dying RPE cells, we next assessed RPE density on phalloidin/IBA1 double-labeled RPE/choroidal flatmounts of 12-month-old Thbs1^−/−^- and Cd47^−/−^-mice (Fig. 4A). Quantifications of RPE cell numbers per square millimeter revealed no significant difference between WT-, Thbs1^−/−^-, and Cd47^−/−^-mice at 12 months, and RPE cell density was similar between WT- and Cd47^−/−^-mice at 6 months of age (Fig. 4B), showing that the accumulation of subretinal melanophages in Cd47^−/−^-mice was not associated with a significant RPE cell loss compared to controls. The age-related MP accumulation in Thbs1^−/−^-, and Cd47^−/−^-mice also revealed no significant loss of photoreceptor cell nuclei rows at 12 months quantified on histological sections (data not shown), contrary to MP accumulation in Cx3cr1^−/−^ and ApoE2-isoform expressing mice. However, while in 6-month-old WT- and Cd47^−/−^-mice and in 12-month-old WT- and Thbs1^−/−^-mice RPE cells were hexagonal in 80% and 60% of cases (the remainder being pentagonal cells), the percentage of hexagonal cells fell to 40% in 12-month-old Cd47^−/−^-mice (Fig. 4C). Conversely, the percentage of dysmorphic RPE cells with less than five or more than six neighbors/sides was significantly elevated in 12-month-old Cd47^−/−^-mice compared to the other strains (Fig. 4A yellow asterixis, Fig. 4D). The vast majority of RPE cells of 12-month-old Cd47^−/−^-mice were fitted with one or two nuclei as in the other strains. However, a small but significant population of RPE cells had three instead of a maximum of two nuclei (Fig. 4E).

In summary, our morphological analysis of the RPE demonstrates that the accumulation of melanophages in Cd47^−/−^-mice is not primarily due to the phagocytosis of dead RPE cells. However, the RPE of 12-month-old Cd47^−/−^-mice had undergone significant morphological changes which might be due to the chronic contact with melanophages.

### CD47-deficient RPE cells lose melanosomes/melanolipofuscin to melanophages

Physiologically, the tips of the outer segments (OS) of the photoreceptors are phagocytosed by the RPE, but when subretinal MPs accumulate, such as with age or in Cx3cr1^−/−^mice, they also phagocytose OS [3, 29]. However, MPs rarely seem to phagocytose melanosome-containing microvilli of the RPE cells, likely due to inhibitory “don’t eat me” signals of the RPE, such as CD47.

**Fig. 5:**
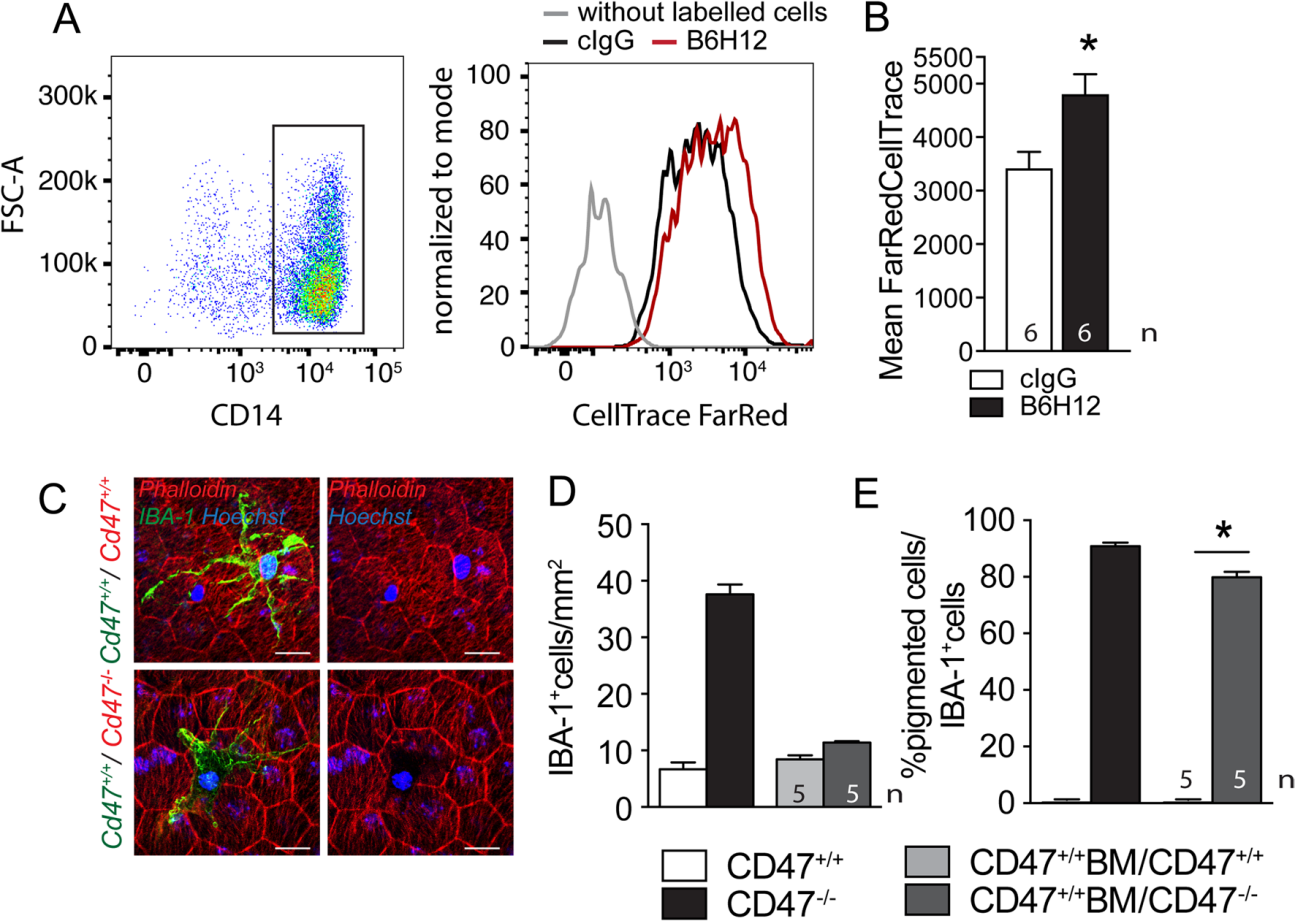
CD47-deficient RPE cells lose melanosomes/melanolipofuscin to melanophages. Gating and FarRed CellTrace intensity measurements by cytometry of human CD14 + Mo after 2 h of incubation with a monolayer of FarRed CellTrace pre-stained ARPE19 cells (a human RPE cell line) with 10 µg/ml of a control antibody (black line) or CD47 blocking antibody B6H12 (red line **A**) and quantification of the fluorescence intensity (**B**; *n* = 6 wells per group from three independent experiments; Mann–Whitney *p* = 0.0221). Three independent experiments gave similar results. Representative micrographs of phalloidin (red fluorescence staining), IBA1 (green fluorescence staining) double-labeled RPE/choroidal flatmounts of 12-month-old Cd47^+/+^ (upper panel) or Cd47^−/−^- recipient mice (lower panel) that had received a Cd47^+/+^- bone marrow transplant after lethal irradiation at 6 months of age (**C**). Quantification of the number of IBA1-stained subretinal MPs (**D**) and quantification of the percentage of pigment-laden melanophages (that block the phalloidin staining of the underlying RPE) of total subretinal MPs (**E**) of 12-month-old WT and Cd47^−/−^-mice compared with Cd47^+/+^ bone marrow transplanted WT and Cd47^−/−^-mice (*n* = 5/group; Mann–Whitney *p* = 0.00,159). Scale bar = 20 µm

To test whether MPs would phagocytose material from living RPE cells, we incubated unlabeled human monocytes with human RPE cell line, ARPE19 cells, that we had previously labeled with FarRed CellTrace (FRCT) either in the presence of a control IgG or the anti-CD47 blocking antibody B6H12. After 2 h of incubation, when no cell death occurs in either RPE or monocytes (data not shown), we observed FarRed Cell Trace uptake in both conditions by flow cytometry, but the population of CD14^+^monocytes had become significantly more FRCT positive when the CD47-blocking antibody was present in the co-culture (Fig. 5A and B), showing that CD47 blockage significantly increases the transfer of FRCT^+^ cytoplasm from ARPE19 cells to monocytes even in this short time period. The forward scatter area (FSC-A, which reflects the cell size) of CD47-blocking antibody treated CD14^+^ FRCT^+^Mos only slightly increased (10–15%) compared to control monocytes (data not shown), demonstrating that monocytes did not phagocytose whole ARPE19 cells (three times the size of the monocyte), but cell parts or vesicles.

Next, to test whether this trafficking would take place in vivo, we transplanted CD47^+/+^ WT bone marrow from mice of a CD45.1 genetic background into 6-month-old lethally irradiated CD47^+/+^- and CD47^−/−^-recipient mice of a CD45.2 genetic background. CD47^−/−^-bone marrow transplanted animals in CD47^+/+^ recipients were not viable confirming previous studies that showed that the transplanted CD47^−/−^-bone marrow gets eliminated by the recipients’ splenic dendritic cells and macrophages [30]. The animals were kept for 6 months after the transplantation to allow the replacement of the retinal microglia by bone marrow-derived cells in this irradiation model without head sparing [31]. At 12 months, 6 months after the lethal irradiation, flow cytometry confirmed the successful engraftment of CD45.1 bone marrow in the recipient mice (data not shown). Phalloidin/IBA1 double-labeled RPE/choroidal flatmounts of the CD47^+/+^BM/CD47^+/+^recipient WT transplanted mice revealed unpigmented subretinal MPs, morphologically akin to the subretinal MPs observed in aged WT- and Thbs1^−/−^-mice (Fig. 5C upper panels). Subretinal MPs in CD47^+/+^BM/CD47^−/−^ recipient chimeras however, were heavily pigmented and blocked the red phalloidin fluorescence of the underlying RPE (Fig. 5C upper panels) similar to the melanophages observed in Cd47^−/−^-mice (Fig. 2). Quantification of the subretinal MP density in both transplanted groups were in a comparable range than 12-month-old WT mice, as would have been expected for the accumulation of CD47^+/+^ MPs. However, quantification of the percentage of melanophages in the subretinal MP population (defined as IBA1^+^ MPs that visibly block the red phalloidin fluorescence of the underlying RPE when viewed in the red channel) revealed that 80% of all subretinal MPs in in CD47^+/+^BM/CD47^−/−^ chimeras were melanophages, comparable to Cd47^−/−^-mice. Melanophages were not observed in CD47^+/+^BM/CD47^+/+^ WT transplanted mice similar to WT mice.

**Fig. 6:**
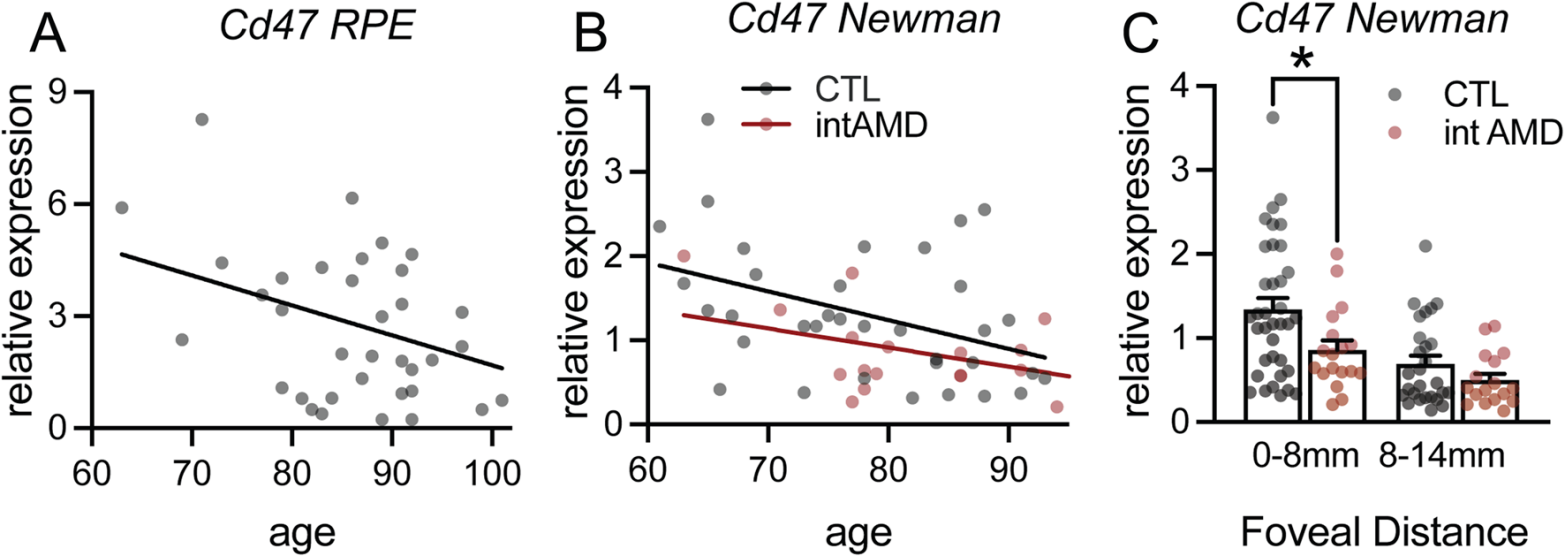
RPE CD47-expression decreases with age and in intermediate AMD in humans. **A** Linear regression of the correlation of age to relative expression of Cd47 mRNA normalized with RPS26 expression in RPE mRNA preparations from 35 subjects older than 60 years determined by quantitative RT-PCR. The subjects had normal post-mortem fundus appearance and no known history of AMD or other retinal diseases (*p* = 0.0261 deviant from zero). **B** Linear regression of the correlation of age of Cd47 mRNA normalized with the average of 40 RPE gene expression in RPE/choroid mRNA preparations from control subjects (CTL *n* = 36; *p* = 0.0156 deviant from zero) and intermediate AMD patients (intAMD *n* = 18; *p* = 0.0704 deviant from zero) from the RPE/choroid transcription data set of [23]. **C** Cd47 mRNA normalized with the average of 40 RPE gene expression in RPE/choroid mRNA preparations from CTL subjects and intAMD patients in central (0-8 mm foveal distance) and peripheral (8–14 mm foveal distance) from the RPE/choroid transcription data set of [23] (*Mann–Whitney *p* = 0.0217)

Together, these experiments reveal that the in vitro inhibition of the CD47-mediated “don’t eat me” signal induces phagocytosis of RPE cells by monocytes and we demonstrate, in vivo, that lack of CD47 on RPE cells is sufficient to induce the accumulation of subretinal melanophages (Fig. 6).

### RPE CD47-expression decreases with age and in intermediate AMD in humans

Our data demonstrate that the vast majority of retinal MCCs in AMD are melanophages. We show that in mice melanophages form due to melanosome/melanolipofuscin transfer from the RPE to subretinal MPs secondary to the reduced expression of the “don’t eat me” signal CD47 on RPE. To assess whether Cd47 expression is altered in AMD, we assessed CD47 expression in RPE and RPE/choroidal mRNA samples of human donors.

Quantitative RT-PCR of macular RPE mRNA preparations from 35 “healthy” subjects older than 60 years revealed that RPE Cd47 mRNA expression significantly decreases with age (Fig. 6A; *p* = 0.0261 deviant from zero), the most important risk factor for AMD. RPE mRNA preparations were obtained by applying 350 µl of RA1 lysis buffer directly to the posterior pole of donor eyes in which we had previously removed the retina. These mRNA preparations are highly enriched for RPE transcripts and contain little choroidal contamination (data not shown). The subjects had normal post-mortem fundus appearance and no known history of AMD or other retinal diseases. Sub analysis for Cd47 mRNA expression levels according to CFH Y402H and the 10q26 variant revealed no influence of these genetic risk factors on Cd47 expression (data not shown).

We next analyzed Cd47 expression in the publicly available data of the transcriptome of RPE/choroid samples of healthy subjects and controls from Newman et al. [23]. We first filtered the data to keep only subjects older than 60 and analyzed the data from the “normal” samples (controls, *n* = 36) and from intermediate AMD patients (*n* = 18) classified in Newman et al. as “MD2” (*n* = 4; soft distinct drusen > 63 µm/pigmentary changes) and “dry AMD” (*n* = 14; soft indistinct drusen > 125 µm, reticular drusen, soft distinct drusen in association with pigmentary changes, soft distinct drusen in association with pigmentary changes). Samples from late AMD patients were not included in the analysis as there were only two to four per subgroup in the dataset. As the amount of RPE mRNA might vary from sample to sample depending on how many viable RPE cells the RPE/choroidal extractions contained, we normalize the expression data of each of the RPE/choroid samples for the content of RPE mRNA using 40 RPE-specific transcripts. These RPE-specific transcripts were selected from the single cell transcriptomic dataset of Voigt et al. [24]. Similar to our RPE mRNA preparations, normalized Cd47 mRNA expression in the central RPE of healthy control subjects diminished significantly with age (Fig. 6B; *n* = 36, *p* = 0.0156 deviant from zero). In intermediate AMD patients, the linear regression line of Cd47 mRNA expression with age was below that of the healthy subjects and did not reach significance (intAMD *n* = 18; *p* = 0.0704 deviant from zero). Last but not least, Cd47 mRNA expression in the Newmann data set revealed a significantly lower expression of CD47 mRNA in the central samples of intAMD patients compared to controls [23] (*Mann–Whitney *p* = 0.0217).

Taken together this data demonstrates that RPE Cd47 transcriptions diminishes with age and in intermediate AMD. This diminished expression of one of the “don’t eat me” signals might promote melanophage formation and associated RPE dysmorphia in AMD where subretinal MPs accumulate.

## Discussion

Not all patients with early/intermediate AMD progress to develop late debilitating AMD and many patients stay stable for years [4, 5]. Hyperreflective foci (HRF) on SD-OCT, provoked by melanosome/melanolipofuscin-containing cells (MCCs) interior to the RPE band [9], have been recognized to be a highly reliable progression biomarker for neovascular AMD and geographic atrophy [6, 25, 32, 33]. The mechanism responsible for AMD progression must therefore cause the appearance of retinal MCCs or MCCs themselves trigger progression to late AMD. It has widely been assumed that MCCs causing HRFs are provoked by RPE cells that migrate into the retina [9, 34]. In a process known as type-2 epithelial–mesenchymal transition (EMT) epithelia from kidney, lung an intestine have been shown to transdifferentiate into mesenchymal cells and participate in fibrosis in inflammatory conditions [35] and a similar transition has been proposed to occur in retinal disease [36]. However, the identification of MCCs as RPE cells in AMD patients is based only on the fact that both contain melanosomes and melanolipofuscin [9], despite the fact that melanosomes, lipofuscin, and melanolipofuscin particles are not specific marker of RPE cells. In retinitis pigmentosa models subretinal macrophages have been beautifully shown to be autofluorescent and to contain melanofuscin particles that are indistinguishable from the RPE [37, 38]. Melanosomes are also produced in melanocytes and can be trafficked from cell to cell. Melanocytes in the skin transfer melanosomes to keratinocytes in the epidermis, but also to macrophages in the normal dermis [28] and in skin disorders [20–22], giving rise to melanophages. Therefore, MCCs, provoking HRF in the retina in AMD, are not necessarily migrating RPE cells. Indeed, our study of sections from 12 eyes with MCCs from 11 AMD patients failed to detect RPE-specific peropsin or RPE65 in any retinal MCCs in any of the sections, but strongly marked all RPE cells in the monolayer even those close to atrophic lesions where RPE cell death occurs. Additionally, we found no evidence that MCCs are positive for GFAP, a marker of astrocytes in the healthy retina and of astrocytes and activated Müller cells in AMD. On the other hand, the majority of MCCs stained positive for MP markers CD68, CD163, and IBA1, which never stained RPE cells integrated in the monolayer and are not known to be expressed by any other retinal or mesenchymal cells, showing a high degree of specificity in this tissue. These results confirm a recent report using similar markers [26]. 70 to 100% of the surface of the histological section that contained pigment was positive for at least one of the MP markers, identifying the majority of MCCs as autofluorescent, melanin-containing MPs, melanophages. In reality the part of melanophages in the MCC population is likely higher as (i) we were not able to stain simultaneously for the three MP markers as they require different substrate incubation times and incompatibilities of secondary antibodies; (ii) MCCs might be negative for our chosen MP markers but positive for others; and (iii) all pigment is not necessarily within cells at all times. It has been argued that the migrating RPE are de-differentiated to a point that they cease to express RPE-specific markers, or that the MCCs positive for MP markers are RPE cells that transdifferentiated into MPs [26, 39]. While RPE cells have been shown to undergo type-2 EMT in vitro, there is no evidence a transdifferentiation into a highly differentiated MP is possible. Keeping in mind that intracellular melanosomes and melanolipofuscin particles are not specific to RPE cells but exist in other cell types and notably in macrophages in the form of melanophages, it seems most plausible that autofluorescent, pigmented cells positive for MP markers (in a condition where MPs infiltrate the tissue) are indeed just that: melanophages.

While melanophages form in the dermis through ingestion of melanosomes from neighboring melanocytes [28], very little is known about the mechanisms of retinal melanophage formation. In GA one might assume that MPs infiltrating the atrophic lesions will phagocytose RPE debris from dead RPE cells, but HRFs, and by extension melanophages, also appear in intermediate- and neovascular-AMD where RPE death is not a prominent feature [32]. These clinical observations raise the question how retinal melanophages form and whether their presence is sufficient to give rise to HRF on SD-OCT. We recently showed that both major genetic AMD risk factors, the CFH H402 variant and a 10q26 haplotype, inhibit TSP1-mediated CD47 activation and subretinal MP elimination, promoting pathogenic inflammation [17, 18]. We here confirm that both aged Thbs1^−/−^- and Cd47^−/−^-mice develop subretinal MP accumulation [17]. However, there was a remarkable difference between the two knockout mouse strains: in Cd47^−/−^-mice the cell bodies of subretinal MPs were bloated with a dense pigment, which SBF-SEM reveals was due to densely packed intracellular melanosomes/melanolipofuscin particles, only rarely observed in Thbs1^−/−^-mice (Figs. 2 and 3) and with striking similarities to melanophages in the dermis of mice [28] and the retinae of AMD patients (Fig. 1). At the same time, the RPE surrounding the melanophages in Cd47^−/−^-mice was markedly less pigmented compared to WT- and Thbs1^−/−^-mice, suggested that melanosomes and melanofuscin particles had been transferred from the RPE to the melanophages. In SDOCT examination, numerous hyperreflective foci adjacent to the RPE line were visible only in Cd47^−/−^-mice characterized by their accumulation of subretinal melanophages. These results confirm experimentally that retinal melanophages can form subretinally and provoke HRFs in SDOCT imaging. The analysis of the RPE monolayer of Cd47^−/−^-mice revealed no age-related, or strain-related change of the RPE cell numbers, suggesting that the melanosomes/melanolipofuscin of the melanophages did not stem from the phagocytosis of dead RPE cells, but were transferred from live RPE cells. However, the percentage of regularly shaped hexagonal RPE cells was significantly reduced to the detriment of irregular shaped RPE and an increase in the variability in size in aged Cd47^−/−^-mice (Fig. 4), a feature also observed in wild-type mice twice the age [40], hyperinflammatory mice [41], and most importantly in intermediate AMD [42]. To date we do not know whether these morphological RPE changes are directly due to the absence of CD47 in the RPE or to the chronic presence of melanophages.

Interestingly, neither Cd47^−/−^-mice, nor Thbs1^−/−^-mice developed photoreceptor degeneration by the age of 12 months, contrary to Cx3cr1^GFP/GFP^- and AMD-risk APOE2-expressing-mice [11, 17, 27, 43]. Cx3cr1^GFP/GFP^-MPs that lack the tonic inhibitory CX3CR1 signaling and APOE2-expressing MPs, overexpress inflammatory cytokines, such as IL1β, which causes rod and cone degeneration [44, 45]. The MPs of Cd47^−/−^-mice and Thbs1^−/−^-mice do not lack the tonic inhibitory signals that can lead to the toxic over-expression of cytokines, but are both deficient in the essential TSP1/CD47 MP elimination signaling, which causes the MP accumulation [17, 18].

CD47, independently of TSP1, functions as the ligand for signal regulatory protein α (SIRPα) [19]. SIRPα is expressed on all MPs and its ligation by CD47 induces a “don’t eat me” signal that inhibits the phagocytosis of the CD47-expressing cell [19]. Melanosomes observed in melanophages of Cd47^−/−^-mice could therefore stem from aberrantly phagocytosed parts of the RPE, such as RPE microvilli to which melanosomes migrate after light onset [46]. Indeed, when we co-cultured monocytes with a FarRed CellTrace pre-stained human RPE cell line for 2 h, we observed a transfer of CellTrace-stained material from the RPE to the monocytes when CD47 was inhibited. Importantly, using CD47^+/+^bone marrow transplantation of CD47^+/+^- and CD47^−/−^- recipients we created a in vivo mouse model where subretinal CD47^+/+^MPs accumulate adjacent to either CD47^+/+^- or CD47^−/−^-RPE cells. As expected, the level of subretinal CD47^+/+^MPs accumulation of around 170 subretinal MPs/eye (10 MPs/mm^2^) in both chimeras was comparable to wild-type mice. However, we only observed melanophages in CD47^−/−^-recipient mice, confirming that the lack of the “don’t eat me” signal in the recipient mice is responsible for the subretinal melanophage-phenotype to occur.

Last but not least, we demonstrate that RPE Cd47 transcription diminishes in human subjects with age, the most important AMD risk factor, and in intermediate AMD compared to control subjects (Fig. 6). This diminished expression of a “don’t eat me” signal on the aging RPE likely promotes melanophage formation and associated RPE dysmorphia if it coincides with subretinal MP accumulation in AMD, similar to our observations in mice.

Interestingly, in aged Cd47^−/−^- and Cd47^−/−^-recipient transplanted mice, melanophages constituted 80% of the subretinal MP population. It is not yet clear, whether the “un-pigmented” 20% of MPs constitute a different subtype of MPs or whether they infiltrated more recently and had not yet acquired the melanophage phenotype. In the dermis it has been shown that pigment of tattoos, which is phagocytosed and kept intracellularly by dermal “pigmented” MPs similar to melanophages, is released upon the death of the pigment-containing MP and taken up by infiltrating MPs that thereby become “pigmented” in a pigment capture–release–recapture cycle [28]. Although purely hypothetical at this stage, a modified pigment capture–release–recapture cycle might take place in the retina: anti-VEGF treatment, which accelerates retinal MP elimination/death in the laser-induced model of neovascular AMD [47], also astonishingly decreases the number of HRFs in patients [32]. If anti-VEGF treatment in patients increased the death of melanophages, the liberated melanin containing particles would be passively transported towards the RPE due to the directional flow of water and ions [48] and could be re-phagocytosed by the RPE. Indeed, RPE cells eagerly take up melanosomes they are in contact with in vitro [49].

The concept that HRFs in AMD are provoked by melanophages, the melanosomes/melanolipofuscin-containing subgroup of MPs that infiltrate the retina in AMD, is also supported by the observation that they are more common in carriers of both major genetic AMD risk factors, the CFH H402 variant and a 10q26 haplotype [8], which we showed promote the accumulation of MPs in the retina [17, 18].

**Fig. 7:**
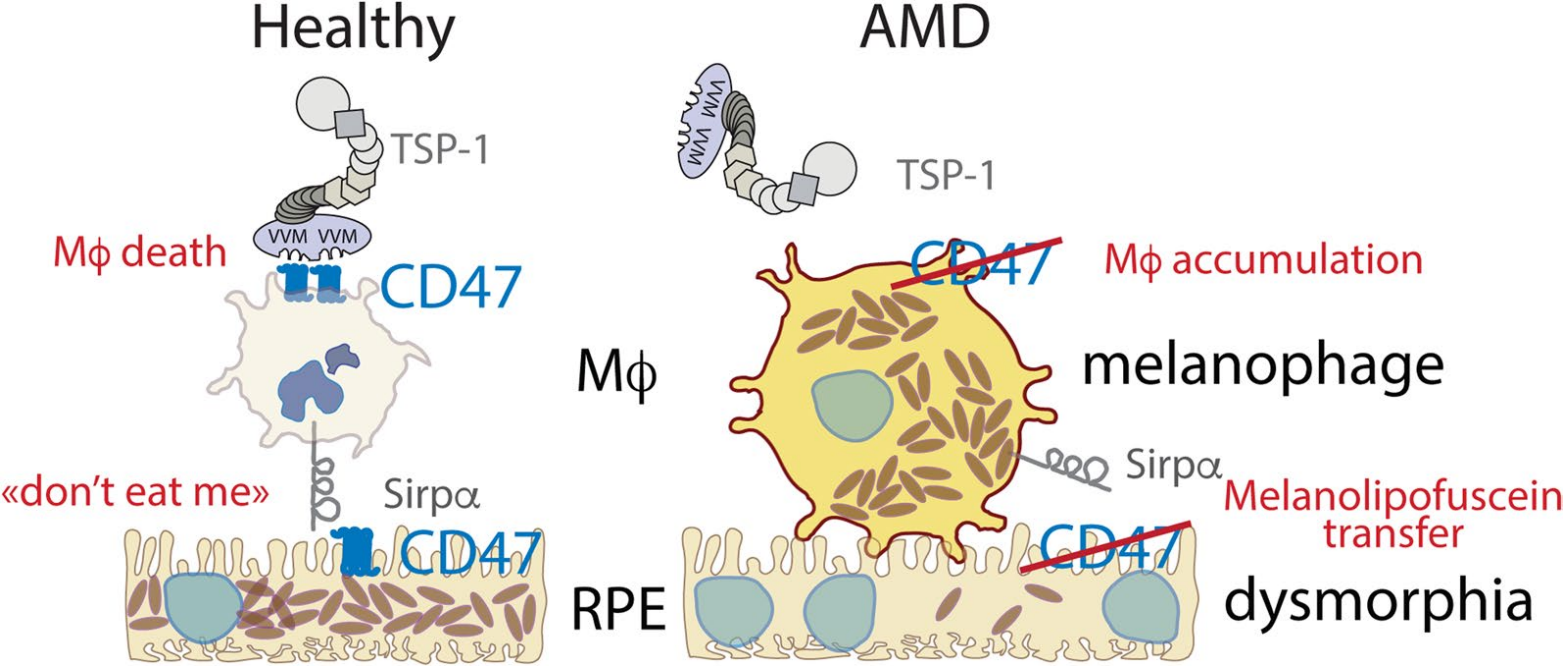
Graphical summary of subretinal melanophage formation in AMD

In summary, our study provides several lines of evidence that retinal melanophages are at the origin of HRFs and shows how they might form in AMD. Our immunohistochemistry on AMD donor eyes demonstrates that MCCs express MP markers but not RPE or macroglial cell markers. We show how retinal melanophages can form in mice in vivo in prior to RPE cell death*,* due to the lack of the CD47 “don’t eat me” signal on RPE cells, and give rise to HRF in SD-OCT. Importantly, we demonstrate that Cd47 transcription diminishes with age and in intermediate AMD, which likely promotes melanophage formation in AMD (Fig. 7). Together, with our previous findings that AMD-genetic risk factors directly promote retinal inflammation [17, 18, 43], our study strongly suggests that retinal melanophages promote AMD progression. HRFs might therefore be a useful biomarker for subretinal MP accumulation in clinical trials. In the future, strategies to increase the expression of “don’t eat me” signals in intermediate AMD might help protect the RPE from collateral damage and prevent progression to blinding late AMD.

CD47 expressed on mononuclear phagocytes (MP) acts as the receptor of the immunosuppressive signal of TSP-1 and mediates subretinal macrophage elimination. CD47 expressed on RPE cells also acts as the ligand of SIRPα, mediating a ‘don’t eat me’ signal. With age and in intermediate AMD CD47 expression on RPE diminishes and we previously showed that TSP1/CD47-dependent elimination is inhibited by the main AMD risk-variants [17, 18]. Similar to CD47^−/−^-mice subretinal macrophages accumulate due to the absence of TSP-1 signaling. Additionally, we show here that the absence of the CD47 ‘don’t eat me’ signal on RPE cells induces the loss of melanosomes to macrophages, RPE dysmorphia, and the formation of melanosome laden macrophages, called melanophages. These melanophages are visible clinically as hyperreflective foci (HRF), a highly predictive imaging biomarker for the progression to late debilitating AMD.

## Supplementary Information

**Additional file 1.** Serial block-face scanning electron microscopy stack and 3D reconstruction of subretinal MP on 12-month-old Thbs1^−/−^-mice. The border of the subretinal MP (green color), and the surface of each melanosome (magenta) were marked on every SBF-SEM section containing the MPs for the three-dimensional reconstruction of melanosome and melanolipofuscin particle distribution in the subretinal MPs; other organelles were marked in white.

**Additional file 2.** Serial block-face scanning electron microscopy stack and 3D reconstruction of subretinal MP on 12-month-old Cd47^−/−^-mice. The border of the subretinal MP (green color), and the surface of each melanosome (magenta) were marked on every SBF-SEM section containing the MPs for the three-dimensional reconstruction of melanosome and melanolipofuscin particle distribution in the subretinal MPs; other organelles were marked in white.

## Data Availability

Not applicable.
